# Co-occurring anthropogenic stressors reduce the timeframe of environmental viability for the world’s coral reefs

**DOI:** 10.1371/journal.pbio.3001821

**Published:** 2022-10-11

**Authors:** Renee O. Setter, Erik C. Franklin, Camilo Mora

**Affiliations:** 1 Department of Geography and Environment, University of Hawai‘i at Mānoa, Honolulu, Hawai‘i, United States of America; 2 Hawaiʻi Institute of Marine Biology, School of Ocean and Earth Science, University of Hawaiʻi at Mānoa, Kāneʻohe, Hawai‘i, United States of America; University of Queensland, AUSTRALIA

## Abstract

Anthropogenic disturbances are posing unprecedented challenges to the persistence of ecosystems worldwide. The speed at which these disturbances reach an ecosystem’s tolerance thresholds will determine the time available for adaptation and conservation. Here, we aim to calculate the year after which a given environmental stressor permanently exceeds the bounds of an ecosystem’s tolerance. Ecosystem thresholds are here defined as limits in a given stressor beyond which ecosystems have showed considerable changes in community assembly and functioning, becoming remnants of what they once were, but not necessarily leading to species extirpation or extinction. Using the world’s coral reefs as a case example, we show that the projected effects of marine heatwaves, ocean acidification, storms, land-based pollution, and local human stressors are being underestimated considerably by looking at disturbances independently. Given the spatial complementarity in which numerous disturbances impact the world’s coral reefs, we show that the timelines of environmental suitability are halved when all disturbances are analyzed simultaneously, as opposed to independently. Under business-as-usual scenarios, the median year after which environmental conditions become unsuitable for the world’s remaining coral reefs was, at worse, 2050 for any one disturbance alone (28 years left); but when analyzed concurrently, this date was shortened to 2035 (13 years left). When analyzed together, disturbances reduced the date of environmental suitability because areas that may remain suitable under one disturbance could become unsuitable by any of several other variables. The significance of co-occurring disturbances at reducing timeframes of environmental suitability was evident even under optimistic scenarios. The best-case scenario, characterized by strong mitigation of greenhouse gas emissions and optimistic human development, resulted in 41% of global coral reefs with unsuitable conditions by 2100 under any one disturbance independently; yet when analyzed in combination up to 64% of the world’s coral reefs could face unsuitable environmental conditions by one disturbance or another. Under the worst-case scenario, nearly all coral reef ecosystems worldwide (approximately 99%) will permanently face unsuitable conditions by 2055 in at least one of the disturbances analyzed. Prior studies have indicated the projected dire effects of climate change on coral reefs by mid-century; by analyzing a multitude of projected disturbances, our study reveals a much more severe prognosis for the world’s coral reefs as they have significantly less time to adapt while highlighting the urgent need to tackle available solutions to human disturbances.

## Abstract 2

An analysis of independent human disturbances shows that, under business-as-usual scenarios, unsuitable environmental conditions will exist for half the world’s remaining coral reefs by 2050 (28 years left), while the combined effects of disturbances yields unsuitable conditions by 2035 (13 years left).

## Introduction

The capacity of ecosystems to persist and potentially adapt to environmental change is primarily determined by the time period in which environmental variables remain suitable [1–6]. Here, we aim to estimate the year after which environmental disturbances permanently exceed tolerance thresholds (i.e., the year after which an environmental variable remains permanently outside suitable conditions). This analysis can be calculated at the species or ecosystem level depending on the availability of tolerance thresholds. As a case example, we applied this concept to coral reef ecosystems under alternative projections of marine heatwaves (degree heating weeks (DHW)), ocean acidification (Ωarag), storms (return time of hurricanes 4 or larger), land-based pollution (as surrogated by human land use), and local human disturbances (as surrogated by human population density). Tolerance thresholds for specific environmental variables were acquired from the literature and are commonly indicative of considerable direct coral reef loss, net functional degradation, and shifts beyond which ecosystems persist only as a remnant or relic that delivers a small fraction of the goods and services it previously provided (see additional details in S1 Text). At the ecosystem level, this exceedance of tolerance thresholds should not necessarily be interpreted as extinction or extirpation of species or ecosystems, but as considerable changes in community assembly and function. The consequences to permanently experiencing unsuitable environmental conditions could be diverse, with several pathways including extinction, adaptation, or alternative ecosystem states (see Methods and S2 Text). The date at which tolerance thresholds were permanently exceeded was estimated at a particular location under alternative scenarios independently for each environmental variable as well as overall environmental unsuitability date as the year at which any of the variables exceeds its tolerance threshold for the remainder of the time series. We analyzed 3 scenarios including the worst-case (business-as-usual emissions and fossil-fueled development [RCP8.5-SSP5]), a mid-case (middle of the road stabilization scenario [RCP4.5-SSP2]), and the best-case (strong mitigation of greenhouse gases and sustainable socioeconomic pathway [RCP2.6-SSP1]) (see Methods and S2 Text).

## Results and discussion

**Fig 1:**
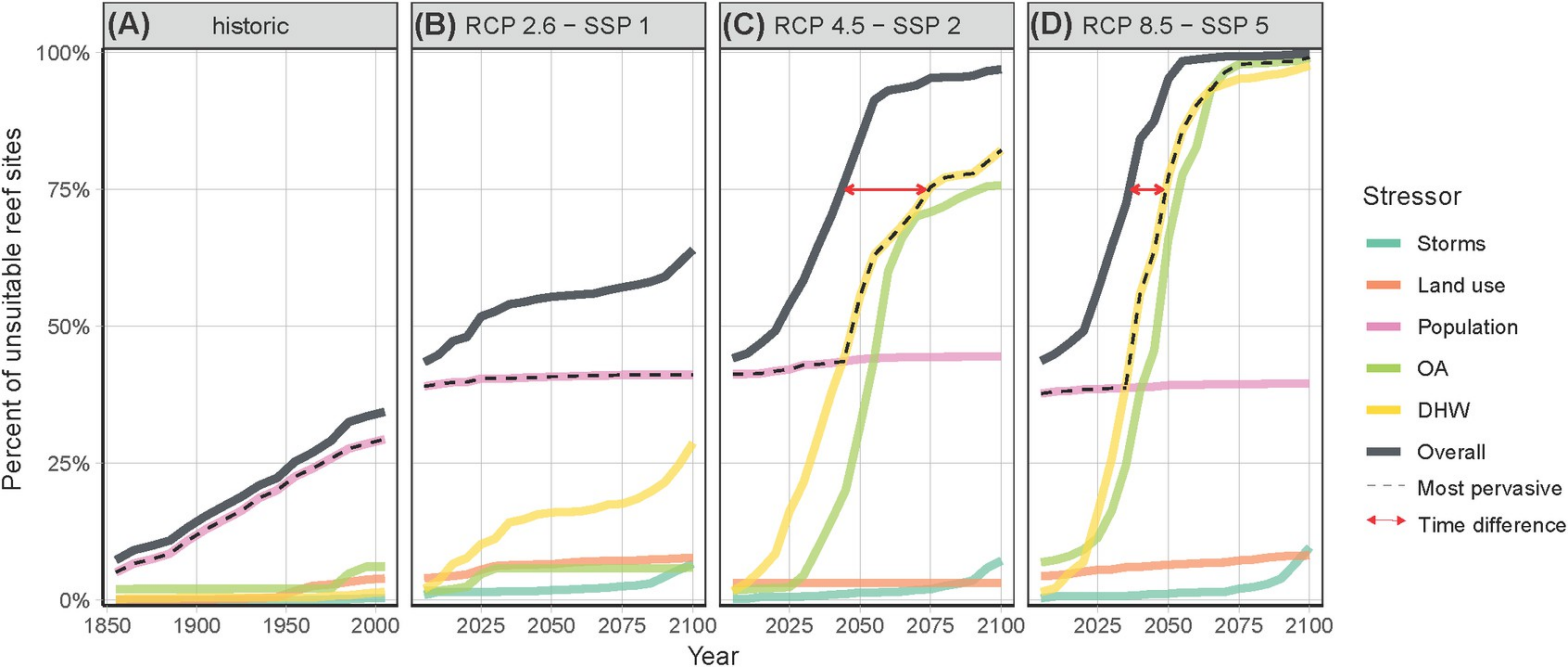
Projected dates of environmental unsuitability for the world’s coral reefs due to disturbances. The cumulative percentage of the world’s coral reefs are displayed by their date of unsuitability due to individual variables (thick colored lines) or in combination when any one stressor experiences an unsuitable condition (overall environmental unsuitability, black line), under historical conditions and 3 RCP-SSP scenarios (A-D). The overall environmental unsuitability refers to cases in which at least 1 out of all 5 studied stressors exceeds its tolerance thresholds. As a reference for shortening of suitability timelines caused by analyzing one or multiple variables, we show the time difference (red arrow) for the unsuitable conditions of 75% of the world’s coral reefs under the most pervasive disturbance alone (dotted black line) and when all available variables are considered, under RCP8.5-SSP5 (i.e., 15 years shortening) and RCP4.5-SSP2 (i.e., 30 years shortening). The data underlying this figure can be found in https://zenodo.org/record/7055724.

Remarkably, we find that by 2005, 44% of the world’s coral reefs already faced unsuitable environmental conditions. While seemingly high, this large area with environmental unsuitability is in line with reported studies on large-scale losses of coral reefs [7–9]. For instance, total coral reef losses upwards of 50% have already been reported for the Caribbean and Indian Ocean [7] while annual declines in live coral cover range from approximately 1% in the Indo-Pacific (over the time period 1997 to 2004) [8] to approximately 1.5% in the Caribbean (over the time period 1977 to 2000) [9]. We find that the primary anthropogenic disturbance driving recent coral reef unsuitability was local-scale pressures related to human population density (Fig 1A). Local human population relates to disturbances such as overfishing, runoff, coastal development, and eutrophication, which have been known to have deleterious impacts on coral reefs [10–13]. The direct pressures of local human populations are particularly severe on coral reefs given their shallow and coastal distribution and the fact that human population has increased dramatically in coastal areas [11,14,15]. Prior studies have already indicated that by 2000, 75% of the world’s coral reefs were adjacent to human settlements [10] with 58% being accessible within less than 30 minutes from the nearest human settlement [12]. Recurring bleaching events of global scope have also added to recent coral degradation [16]. For instance, approximately 16% of the world’s coral reefs died due to single bleaching events like those occurring in 1998 and 2016 [17].

No individual disturbance was completely responsible for the environmental unsuitability of coral reefs, but when the stressor effects were considered together, they resulted in broader and earlier dates of environmental unsuitability. This was due to the existence of multiple spatially complementary stressors that caused reefs free of one stressor to face unsuitable conditions from another; as a result, not only a larger area of reefs was under risk, but the timeframe to exposure of continuously unsuitable conditions in at least one of several disturbances occurred sooner. As indicated earlier, considerable environmental unsuitability has already occurred due to localized human drivers, particularly along continental masses and tropical regions (S4 and S5 Figs). Going forward, environmental unsuitability by heatwaves is projected to occur first in tropical areas and later at higher latitudes (S2 Fig), while environmental unsuitability by acidification is projected to occur first in higher latitudes and later at tropical areas (S3 Fig; see also [6,18]). Environmental unsuitability by storms spreads earlier in pantropical areas towards higher latitudes and the tropics (S6 Fig). The spatial complementarity between heatwaves, acidification, storms, and local human disturbances increased the global extent in which environmental conditions became unsuitable for coral reefs, but also considerably shortened the dates of suitable environmental conditions.

**Fig 2:**
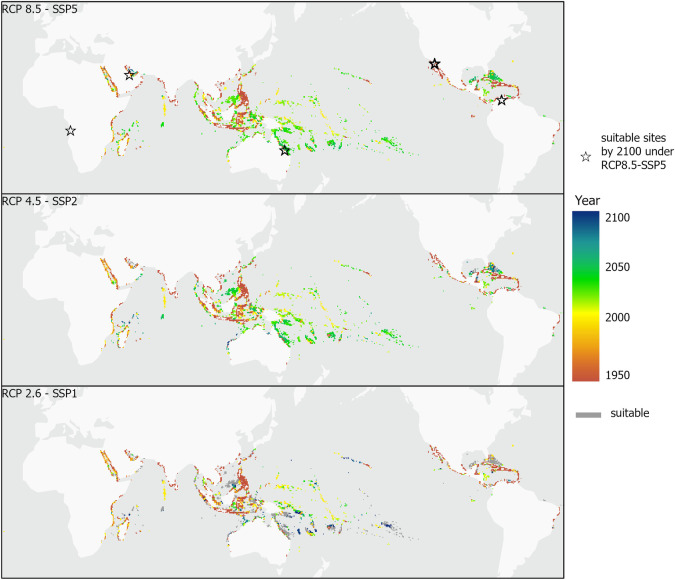
Coral reef dates of unsuitability by projected human and environmental disturbances. A global map of the date after which optimal environmental conditions are permanently exceeded for coral reef ecosystems under each scenario (RCP8.5-SSP5, RCP4.5-SSP2, and RCP2.6-SSP1). The map illustrates the first year at which suitable conditions cease to exist due to marine heatwaves, ocean acidification, storms, land use, or local human stressors. Stars highlight shallow reef sites that, by 2100 under the worst-case scenario (RCP8.5-SSP5), might escape the array of human disturbances analyzed in our study. Sites indicated in grey remain suitable by 2100. The data underlying this figure can be found in https://zenodo.org/record/7055724. Basemap provided by Esri [53].

**Fig 3:**
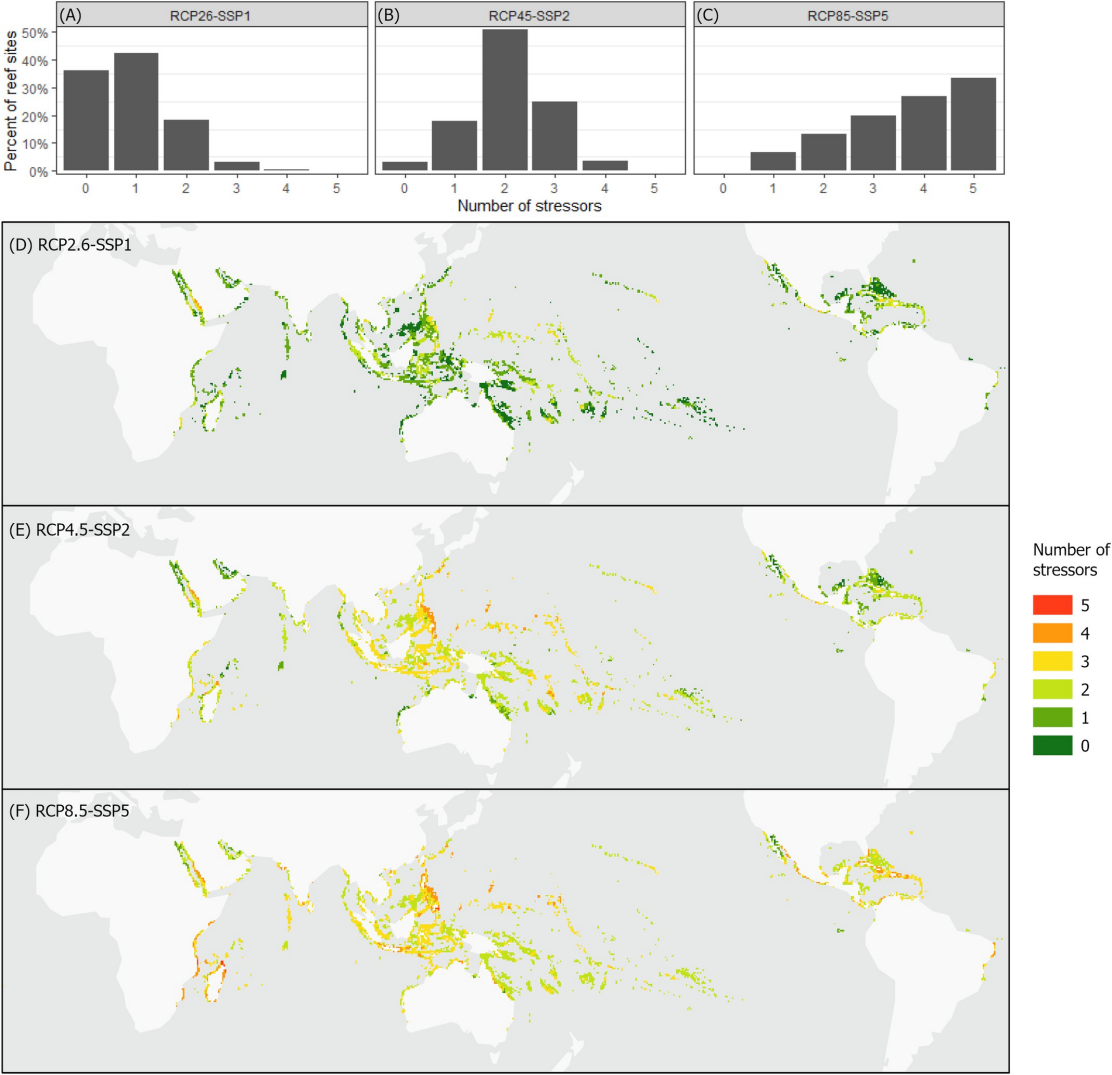
World’s coral reefs by the number of stressors exceeding ecosystem tolerance thresholds. Plots A, B, and C show the percentage of the world’s coral reefs at 2100 by the number of stressors exceeding tolerance thresholds. Plots D, E, and F illustrate the same data spatially. The data underlying this figure can be found in https://zenodo.org/record/7055724. Basemap provided by Esri [53].

The reef areas with unsuitable conditions increased and the timelines with suitable conditions reduced under simultaneous, as opposed to independent, disturbances because areas that may have remained suitable under one disturbance became unsuitable by any of several other stressors (Fig 1). Under the worst-case scenario, for the world’s coral reefs remaining viable in 2005, the soonest average date of environmental unsuitability by any one disturbance alone was 2055. But when all available stressors were considered simultaneously (i.e., environmental unsuitability could be caused by any of several disturbances), the average coral reef was projected to face unsuitable conditions by 2035 by one variable or another (for reference, see red line in Fig 1D). When environmental unsuitability could be caused by any of the disturbances considered, unsuitable conditions for at least one variable would occur in 64% of the world’s coral reefs by 2100 under the best-case scenario, while nearly all coral reefs (approximately 99%) would experience unsuitable conditions by 2055 under the worst-case scenario (Figs 1 and 2). By 2100, 93% of the world’s coral reefs will face more than one unsuitable environmental condition under RCP8.5-SSP5, and 22% under RCP2.6-SSP1 (Fig 3). Unfortunately, under the simultaneous effects of all analyzed human disturbances in worst-case scenarios, most rocky reef sites outside the current distribution of coral reefs were also found to lose environmental viability due to waters being too acidic or hot for coral survival (Fig 2). While this study does not account for adaptation potential (see S2 Text), it is clear that limited opportunities exist for dispersal and assisted migration as mechanisms for escaping future environmental stress (under the worst-case scenario, only a few locations were found to remain suitable by 2100 in the Persian Gulf, Red Sea, Baja California, West Africa, Caribbean Venezuela, and Northern Great Barrier Reef). The immense area of reefs at risk and narrower timelines of environmental suitability, under multiple, simultaneously occurring human disturbances, combined with a limited set of areas that can work as environmental refugia, pose a considerable challenge for coral reef ecosystems to adapt while highlighting the urgency to implement mitigation and conservation efforts.

The shorter timeline of suitable environmental conditions when analyzing multiple disturbances simultaneously was also evident even under optimistic scenarios. For instance, RCP2.6-SSP1 is intended to describe the most aggressive mitigation of greenhouse gases and most optimistic scenarios of human development; independently, disturbances under this scenario yield unsuitable conditions for 41% of the world’s coral reefs by 2100, yet when all disturbances were considered simultaneously, by the same year, 64% of the world’s coral reefs faced unsuitable environmental conditions in at least one of the several disturbances analyzed (Fig 1B). Similarly, RCP4.5-SSP2 describes some degree of mitigation of disturbances as opposed to business-as-usual under RCP8.5-SSP5. While the mitigation under the former scenario leads to a smaller area of the world’s coral reefs surpassing thresholds under independent stressors, the combination of disturbances leads to a similarly large area of the world’s coral reefs with unsuitable environmental conditions due to any of the several disturbances studied under either mitigation scenario [i.e., by 2100, approximately 97% (for RCP4.5-SSP2) and approximately 99% (for RCP8.5-SSP5) of the world’s coral reefs would face unsuitable conditions by any of the stressors studied; Fig 1C and 1D].

Considerable attention has been given to the issue of climate change, especially as it relates to changes in temperature and acidification on coral reefs [6,18,19]. These studies all highlighted a dire prognosis for the world’s coral reefs around the middle of the century (e.g., significant environmental crises have been projected to occur from 2050 to 2080 under projected changes of temperature and/or acidification [6,18–21]). While concurring with these prior results, our study suggests that assessing suitability from too few variables might underestimate the dangers of human disturbances on coral reefs, as supported by studies investigating effects of cumulative stressors at a local scale [22–26] and studies on planetary and biodiversity boundaries [27,28]. As shown in Fig 1, under the business-as-usual scenario, the time remaining with suitable conditions for 75% of the world’s coral reefs shortens from 2050 (28 years left) under any individual stressor to 2035 (13 years left) when the effects of all stressors were considered simultaneously. This suggests that the time available for coral reefs to cope with human disturbances is half what has been previously thought. Clearly, ecosystems are running out of time to adapt to human disturbances, compounded with a reduced number of viable areas for expansion beyond their current distribution. While a few locations might escape the multitude of human disturbances and should be the focus of direct management efforts, our results indicate that isolated conservation projects might prove futile and create a false sense of security as sites may offer refugia from some but not all disturbances [29,30]. Our results should not discourage policymakers and conservation efforts but rather increase the sense of urgency towards rapidly curtailing greenhouse gas emissions and investment in conservation in order to avoid further degradation of coral reef ecosystems. Previously, it has been suggested that the long-term persistence of coral reefs will require strategic management actions informed by multiple social-environmental drivers [16,31]; our paper reveals the extremely short timeline in which these would need to be implemented.

## Methods

In this study, we calculate the year after which the values of a given disturbance permanently exceed the variable’s tolerance threshold for a given ecosystem. We applied this analysis to the world’s coral reefs as a case example. Thresholds were obtained from the scientific literature and commonly reflect levels at which specific variables have caused considerable loss of coral reefs leading to changes in community assembly and ecosystem functioning. In our analysis, we considered the year after which such thresholds were permanently exceeded, thus indicating the remaining timeframes for suitable environmental conditions. The expected consequences of permanently experiencing unsuitable environmental conditions could be diverse, including extinction and/or extirpation, adaptation and the implied loss of non-suited genotypes, and/or modification of community assembly and function as it has been observed in many modern coral reefs that have transformed from “coral-covered” states to reefs dominated by macroalgae or, at times, bivalves, sponges, tunicates, zoanthids, or octocorals when faced with unprecedented environmental conditions [32]. Thresholds could not be standardized among disturbances by a common response, in part due to the lack of such comprehensive studies, but also because stressors can vary in the way they affect ecosystems [32]. Nevertheless, all thresholds used indicated conditions beyond which have been known to cause deleterious or transformative effects on ecosystems. The variables analyzed included marine heatwaves (DHW), ocean acidification (Ωarag), storms, land-based pollution (as surrogated by human land use), and local human disturbances (as surrogated by human population density; see S1 Text for caveat regarding additional stressors). These last 2 variables are proxies commonly used to represent other human disturbances that are known to affect coral reefs, such as fishing, eutrophication, dredging, coral mining, vessel impacts, etc. [11,15]. The thresholds for these variables and the references from where they were obtained are listed in Table 1. Detailed methods on how variables were calculated and thresholds used are presented in Extended Methods in S1 Text.

**Table 1:** Variable suitability thresholds. Thresholds used to define suitability for each variable including degree heat weeks (DHW), Ω aragonite, storms, population density, and land use. Additional details on the underlying data and metrics are indicated in the Extended Methods section (see S1 Text for caveats regarding threshold validation and variations in thresholds).

Variable	Ideal values	Reef changes under threshold exceedance	Source
**Degree heat weeks**	<8 DHW	Marine heatwaves and repeated, sustained amounts of high sea surface temperatures are commonly linked to coral bleaching events.	[18,33,34]
**Ω aragonite**	Ω aragonite >3.3	Field studies show that as acidity increases and Ω aragonite decreases, reef accretion approaches zero or becomes negative.	[19,35,36]
**Storm**	Storm strength <category 4 Return period >5 years	Strong storms have been linked to considerable damage on reefs, while short storm return period prevents coral reef recovery.	[37–39]
**Population density**	Log population density <2 in a 50-km radius around reef	High population density correlated with coral growth anomalies and disease, low biodiversity and fish biomass, and lower community trophic level.	[10,11,14,15,40–45]
**Land use**	Summed proportion agricultural/urban land use <0.5 in a 50-km radius around reef	Terrestrial runoff due to agricultural and coastal urbanization can lead to increased levels of sedimentation and eutrophication, leading to decreased colony sizes, growth anomalies, and reduced growth and survival.	[46–50]

Temporal data for each of the environmental stressors was obtained globally starting in 1850 to 2005 to represent the historical time period, and from 2005 to 2100 to represent 3 alternative future case scenarios. The best-case scenario included a strong mitigation of greenhouse gases [Representative Concentration Pathway 2.6 (RCP2.6)] and a sustainable socioeconomic pathway [Shared Socioeconomic Pathway 1 (SSP1)], while the worst-case scenario included business-as-usual emissions (RCP8.5) and fossil-fueled development (SSP5); the middle scenario included a stabilization emissions experiment (RCP4.5) and a middle-of-the-road socioeconomic development (SSP2). Sources used for each variable are outlined in S1 and S2 Tables. The different variables were extrapolated to a common 1 × 1 degree grid using bilinear interpolation and averaged over 5-year time windows. Databases were available at different temporal resolution ranging from every month to every decade; we averaged these variables into 5-year time windows to standardize among variables. Earth System Models are known to have a bias (i.e., deviation from actual conditions) depending on the initial parameters in the models [51]. This bias can affect the results of threshold-based approaches like our analysis as the threshold might or might not be exceeded simply as a result of the added magnitude of this bias. To account for this error, for each climatic variable (i.e., temperature, acidification, storms), we calculated a pixel-by-pixel bias correction, which was the difference between the Earth System Model variable and empirical data during the historical periods in common between the two. This bias correction was specific to each Earth System Model and was added or subtracted to the projections of the given model before conducting analysis.

To calculate the date at which tolerance thresholds were exceeded, we started by assessing the suitability for each variable, which was simply binary values (0 and 1) depending on if the variable exceeded the threshold of suitability or not; a given cell was assigned a 0 value to denote unviable conditions and 1 to indicate suitable conditions (see Table 1). For a given cell, the year of tolerance threshold exceedance was calculated as the first year when the given variable became permanently unsuitable (i.e., remained unsuitable until 2100). These calculations were done independently for each variable and the results are presented as colored lines in Figs 1 and S2–S6 (see S2 Text for caveats regarding stressor interaction). We also calculated an overall environmental unsuitability as the earliest year that any stressor permanently crosses its tolerance threshold and becomes unsuitable in a given cell (Figs 1 [black line] and 2 [all scenarios]).

The global distribution of coral reefs was based on the UNEP-WCMC database (see S1 Table) and additional rocky reef sites from Halpern and colleagues [14,52]. The polygon shapefiles were reprojected into an equal area projection and converted to 1 km grid. The values for different environmental variables were collected at the center of each reef cell.

## Supporting information

S1 Text Extended Methods. (DOCX)

S2 Text Caveats. (DOCX)

S1 Table Data sources. (DOCX)

S2 Table CMIP5 models. (DOCX)

S1 Fig Coral and rocky reef locations. (DOCX)

S2 Fig Date of unsuitable conditions by DHW. (DOCX)

S3 Fig Date of unsuitable conditions by Ωarag. (DOCX)

S4 Fig Date of unsuitable conditions by local human stressors (as surrogated by human population density). (DOCX)

S5 Fig Date of unsuitable conditions by land use. (DOCX)

S6 Fig Date of unsuitable conditions by storms. (DOCX)

S7 Fig Sensitivity analysis to variations in threshold values. (DOCX)

## Abbreviations

DHW: degree heating weeks
RCP: Representative Concentration Pathway
SSP: Shared Socioeconomic Pathway
