# Supplementary material for: Co-occurring anthropogenic stressors reduce the timeframe of environmental viability for the world’s coral reefs
Source: PLoS Biol. 2022 Oct 11;20(10):e3001821. doi: 10.1371/journal.pbio.3001821 (PMC9553053; doi:10.1371/journal.pbio.3001821)
Supplement: S1 Text — (DOCX) [file pbio.3001821.s001.docx]

## **S1 Text. Extended Methods.**

Projected CMIP5 environmental data (SST, salinity, total alkalinity, total dissolved inorganic CO2) was collected through ESGF-CoG (see Table S1 for list of data sources and Table S2 for list of models used). Datasets were remapped from curvilinear to latlon using Climate Data Operator (https://code.mpimet.mpg.de/projects/cdo/; see S1 Text for caveat regarding data resolution).

Data on Sea Surface Temperature from Earth’s System Models (ESM) were bias-corrected to the empirical dataset (see data source in S1 Table); for this, data from each ESM and the empirical data were averaged for the period between 1982 to 2005, then the difference between each model mean and the mean empirical data was calculated and assumed as the bias in the given model; that bias was then added or subtracted to all temperature data of the given ESM, and used for the calculation of Degree Heating Weeks (DHW). DHWs were calculated independently for each ESM using the methodology of prior studies [1–3] wherein the maximum monthly mean (MMM) was calculated as the maximum monthly SST between 1982 and 1992, then the DHW was calculated by finding the difference between a given monthly value and the MMM for each cell. Monthly values were transformed to weeks by multiplying by 4.34, then summed to obtain annual DHW values. This procedure was done independently on each ESM, then the multi-model median was used for calculation of date of environmental unsuitability. The threshold used was 8 degree heating weeks, which has been used in previous threshold refugia studies and is commonly used to predict severe bleaching events [1–3]. In this study, any sites with values exceeding 8 DHW were considered unsuitable.

Ωarag (an indicator of ocean acidification) was calculated based on the inputs of average salinity, SST, total alkalinity, total dissolved inorganic carbon, total phosphate, and total silicate using the method provided by CO2Sys-Excel [4]. Raw dataset values were input into CO2Sys and then the bias correction was calculated on those resulting omega aragonite values. Models were bias corrected to the empirical data, then the multi-model median was calculated and used for analysis. Focal statistics were utilized in order to obtain values for coastal regions – the mean of the eight neighboring cells were used to calculate missing coastal values. The threshold used was Ω aragonite of 3.3, which has been used as a threshold in prior ocean acidification modeling studies and in field studies that show reef accretion approaches zero or becomes negative beyond this threshold [5–7]. In this study, any sites with values less than 3.3 were considered unsuitable.

Since no global datasets are available that project sewage/eutrophication, fishing, dredging, coral mining, vessel impacts, etc., proxies were used to represent the impact that direct/localized human activity has on coral ecosystems. It is challenging to quantify human impacts on neighboring reef ecosystems, nevertheless human population density has been found to have a negative correlation with biodiversity and fish biomass [8,9]. Human population density, while not a perfect match for measuring overall local human impacts on marine environments, is the closest indicator available to understand local stressors. Data on gridded human population density was not available in a single database over the full period from 1850 to 2100 as analyzed for the other variables. Historical data on human population density from 1850 to 2005 were obtained from HYDE [10], while future projections were obtained from IAM [11,12]. Future human density projections were obtained under Shared Socioeconomic Pathways (SSP) 1, 2, and 5, which previously have been paired with emission scenarios RCP2.6, RCP4.5 and RCP8.5, respectively (35). Historical human population data are already calculated as density, but future projections are only available as human population counts. To standardize among datasets, future projections of human population density were calculated by dividing available gridded population counts by the area of the grid cell. At each coral reef pixel, human population density was estimated as the maximum human population density within a 50 km buffer around the grid-cell mid-point. The threshold used was a log population density of 2, as this density has been found in previous studies to be correlated with growth anomalies and negative effects on reef systems and has been used in previous studies as a proxy for local human impacts [8,9,13–15]. In this study, any reef site exceeding log human population density of 2 in a 50km radius around the given reef site was considered unsuitable.

Changes in land cover are known to have deleterious impacts on coral reefs [16–20]. Directly, landcover changes can lead to sedimentation, while land used for croplands, livestock, and urban areas can lead to nutrient and sewage runoff and pollution, triggering eutrophication and adding competitive advantages for algal growth. In this paper, we used the Harmonized Global Land Use database [21], which projects human population under several scenarios, but does not account for potential impacts of climate change leading to alternative population outcomes. The threshold for land cover was when a pixel within a 50km buffer around a given reef site had 50% or more of the area covered by agricultural and urban land. Land areas near reefs that have lost over 50% of their natural land cover have been shown to have increased levels of sedimentation and leave coral more susceptible to bleaching, disease, or mortality [18,20]. In this study, any reef with a land pixel within a 50km radius with 50% or more agricultural and urban land was defined as unsuitable.

Storm impact was quantified by the return period of severe storms. The threshold for storms was conservatively defined where increasing storm intensity and frequency leads to more damage to reefs [22–24], so the threshold for unsuitability was defined as a cell with repeat severe storms (category 4 and greater, or > 113 knots) occurring within a 5-year return time. The only available projection of storms under future emissions scenarios is that by Emanuel based on the power dissipation index [25,26]. The power dissipation index calculates the sum of the maximum wind speed cubed and is available for CMIP5 scenarios. An equivalence of the power dissipation index to storm intensity was calculated by using the quantile of severe storm (category 4) and the equivalent quantile power dissipation index (PDI) value. The return year of extreme storms was calculated as the number of years between repeat storms with PDI analogous to those of storms of category 4 or larger. The threshold used was category 4 severity with a return period of 5 years, as this severity has been linked to considerable damage on reefs while such a high frequency prevents coral reef recovery [22–24]. In this study, any site with a return period less than 5 years of severe storm (category 4 and greater) was considered unsuitable.

Reef site dates of unsuitable conditions were determined for each stressor individually at 10-year intervals under the historical time period and in 5 year intervals under future scenarios by comparing stressor values to their thresholds (see Table 1 for threshold values). Overall unsuitable conditions were then determined at each site and time interval where a site is unsuitable if any individual stressor has exceeded tolerance thresholds at that site. Reef site locations with associated dates of unsuitable conditions were then converted into point shapefiles and visualized in ArcGIS Pro.

# **References**

1. van Hooidonk RJ, Maynard JA, Manzello D, Planes S. Opposite latitudinal gradients in projected ocean acidification and bleaching impacts on coral reefs. Glob Change Biol. 2014;20: 103–112. doi:10.1111/gcb.12394

2. van Hooidonk RJ, Huber M. Quantifying the quality of coral bleaching predictions. Coral Reefs. 2009;28: 579–587. doi:10.1007/s00338-009-0502-z

3. Wellington GM, Glynn PW, Strong AE, Navarrete SA, Wieters E, Hubbard D. Crisis on coral reefs linked to climate change. Eos Trans Am Geophys Union. 2001;82: 1–5. doi:10.1029/01EO00001

4. Environmental Sciences Division ORNL. MS Excel Program Developed for CO2 System Calculations. Carbon Dioxide Information Analysis Center (CDIAC); 2012. doi:10.3334/cdiac/otg.co2sys_xls_cdiac105a

5. Hoegh-Guldberg O, Mumby PJ, Hooten AJ, Steneck RS, Greenfield P, Gomez E, et al. Coral reefs under rapid climate change and ocean acidification. Science. 2007;318: 1737–1742. doi:10.1126/science.1152509

6. Kleypas JA, Mcmanus JW, Meñez LAB. Environmental limits to coral reef development: Where do we draw the line? Am Zool. 1999;39: 146–159. doi:10.1093/icb/39.1.146

7. Kleypas J, Langdon C. Coral reefs and changing seawater carbonate chemistry. Coral Reefs Clim Change Sci Manag Coast Estuar Stud. 2006;61. doi:10.1029/61CE06

8. Mora C, Aburto-Oropeza O, Bocos AA, Ayotte PM, Banks S, Bauman AG, et al. Global human footprint on the linkage between biodiversity and ecosystem functioning in reef fishes. PLOS Biol. 2011;9: e1000606. doi:10.1371/journal.pbio.1000606

9. Cinner JE, Graham NAJ, Huchery C, Macneil MA. Global effects of local human population density and distance to markets on the condition of coral reef fisheries. Conserv Biol. 2013;27: 453–458.

10. Klein Goldewijk K, Beusen A, Van Drecht G, De Vos M. The HYDE 3.1 spatially explicit database of human-induced global land-use change over the past 12,000 years: HYDE 3.1 Holocene land use. Glob Ecol Biogeogr. 2011;20: 73–86. doi:10.1111/j.1466-8238.2010.00587.x

11. Jones B, O’Neill BC. Spatially explicit global population scenarios consistent with the Shared Socioeconomic Pathways. Environ Res Lett. 2016;11: 084003. doi:10.1088/1748-9326/11/8/084003

12. Gao J. Downscaling Global Spatial Population Projections from 1/8-degree to 1-km Grid Cells. NCAR Library; 2017 Aug. doi:10.5065/D60Z721H

13. Halpern B, Walbridge S, Selkoe KA, Kappel CV, Micheli F, D’Agrosa C, et al. A global map of human impact on marine ecosystems. Science. 2008;319: 948–952.

14. Aeby GS, Williams GJ, Franklin EC, Haapkyla J, Harvell CD, Neale S, et al. Growth Anomalies on the Coral Genera Acropora and Porites Are Strongly Associated with Host Density and Human Population Size across the Indo-Pacific. Voolstra C, editor. PLoS ONE. 2011;6: e16887. doi:10.1371/journal.pone.0016887

15. Aeby GS, Williams GJ, Franklin EC, Kenyon J, Cox EF, Coles S, et al. Patterns of Coral Disease across the Hawaiian Archipelago: Relating Disease to Environment. Voolstra CR, editor. PLoS ONE. 2011;6: e20370. doi:10.1371/journal.pone.0020370

16. Mora C. A clear human footprint in the coral reefs of the Caribbean. Proc R Soc B Biol Sci. 2008;275: 767–773. doi:10.1098/rspb.2007.1472

17. Packett R, Dougall C, Rohde K, Noble R. Agricultural lands are hot-spots for annual runoff polluting the southern Great Barrier Reef lagoon. Mar Pollut Bull. 2009;58: 976–986. doi:10.1016/j.marpolbul.2009.02.017

18. Maina J, Moel H de, Zinke J, Madin J, McClanahan T, Vermaat JE. Human deforestation outweighs future climate change impacts of sedimentation on coral reefs. Nat Commun. 2013;4: 1–7. doi:10.1038/ncomms2986

19. Prouty NG, Cohen A, Yates KK, Storlazzi CD, Swarzenski PW, White D. Vulnerability of coral reefs to bioerosion from land-based sources of pollution. J Geophys Res Oceans. 2017;122: 9319–9331. doi:10.1002/2017JC013264

20. Kroon FJ, Schaffelke B, Bartley R. Informing policy to protect coastal coral reefs: Insight from a global review of reducing agricultural pollution to coastal ecosystems. Mar Pollut Bull. 2014;85: 33–41. doi:10.1016/j.marpolbul.2014.06.003

21. Chini LP, Hurtt GC, Frolking S. Harmonized Global Land Use for Years 1500 -2100, V1. Data Set. 2014 [cited 19 Feb 2021]. doi:10.3334/ORNLDAAC/1248

22. Gardner TA, Côté IM, Gill JA, Grant A, Watkinson AR. Hurricanes and Caribbean coral reefs: Impacts, recovery patterns, and role in long-term decline. Ecology. 2005;86: 174–184. doi:10.1890/04-0141

23. Puotinen M, Maynard JA, Beeden R, Radford B, Williams GJ. A robust operational model for predicting where tropical cyclone waves damage coral reefs. Sci Rep. 2016;6: 26009. doi:10.1038/srep26009

24. Puotinen M, Drost E, Lowe R, Depczynski M, Radford B, Heyward A, et al. Towards modelling the future risk of cyclone wave damage to the world’s coral reefs. Glob Change Biol. 2020;26: p.4302-4315. doi:10.1111/gcb.15136

25. Emanuel KA. Downscaling CMIP5 climate models shows increased tropical cyclone activity over the 21st century. Proc Natl Acad Sci. 2013;110: 12219–12224. doi:10.1073/pnas.1301293110

26. Emanuel K. Response of global tropical cyclone activity to increasing CO2: Results from downscaling CMIP6 models. J Clim. 2021;34: 57–70. doi:10.1175/JCLI-D-20-0367.1
