# Supplementary material for: Co-occurring anthropogenic stressors reduce the timeframe of environmental viability for the world’s coral reefs
Source: PLoS Biol. 2022 Oct 11;20(10):e3001821. doi: 10.1371/journal.pbio.3001821 (PMC9553053; doi:10.1371/journal.pbio.3001821)
Supplement: S2 Text — (DOCX) [file pbio.3001821.s002.docx]

## **S2 Text. Caveats.**

There are some limitations to this study.

**Data resolution:** Due to the coarse resolution of CMIP5 environmental projections, there is a risk of generalizing the characteristics that may not be representative of the entire area in a reef pixel. The coarse resolution impacts our ability to accurately assess the impacts from each environmental disturbance at a resolution that may be relevant to coral reefs. This caveat cannot be overcome as the global environmental projection data is only available at this coarse resolution. We recommend for future studies an improved resolution in global circulation models and downscaling at a global scale to see the full effect of finer resolution data on these results. That being said, some studies have indicated that changes predicted at coarse spatial scale are still predicted to occur at finer spatial scales [1], and certainly the general patterns observed at coarse resolution are unlikely to be reversed by using finer resolutions.

**Additional stressors**: In this paper, we studied five of the most pervasive known stressors to coral reefs. Certainly, there are additional disturbances that could be included in our analysis (e.g., salinity, turbidity) and lead to changes in our results. However, adding additional stressors most likely would increase the chances that some pixels would experience earlier dates of unsuitable conditions, suggesting that our results are conservative.

**Stressor interaction**: In our analysis, we assume that tolerance thresholds to individual variables are not influenced by other variables. However, the extent to which ecological responses vary when variables interact could affect our results depending on the nature of the interaction. In cases where interacting variables reduce ecosystem tolerances (e.g., synergistic interactions [2]) our analysis could be underestimating dates of unsuitable conditions as actual thresholds may be much lower when variables interact. The opposite effect on the analysis will occur when variables are antagonistic [3], as tolerance thresholds may be higher when variables interact. Cases where variables interact additively [3] should not have an effect on our results. These alternative effects of interacting variables in the results of our analysis were not considered given the large number of possible threshold combinations under interacting variables, and should thus be considered as a limitation of our study.

**Ecosystem vs species**: Our analysis was restricted to the ecosystem level mostly because of the existence of generally accepted and peer-reviewed thresholds that lead to the loss of coral reefs. The analysis could potentially be deployed at the species level but we lack much of the vast amount of species-specific data about tolerance thresholds to numerous environmental variables.

**Uncertainty**: It is common for studies using Earth System Models to provide an indication of the future uncertainty by quantifying the variability among numerous models. While that is possible for individual variables like temperature, acidification, and storms, which are available for several models, such a diversity of outputs was not available for human population density and land use changes, which came with only one output. This situation prevented us from calculating measurements of uncertainty within case scenarios.

**Threshold validation**: The thresholds used have been defined in the scientific literature and are based on empirical data that has been collected across the world. Unfortunately, due to the number of variables analyzed, a field validation of all thresholds would require an incomprehensible amount of field sampling over time in order to properly represent every combination across all environmental disturbances. While the lack of validation of thresholds in many areas of the world should be considered as limitation of our study, we carried out a sensitivity test (described below) in which increases and decreases in the thresholds used resulted in relatively minor effects on the years after which the disturbances in combination caused permanently unsuitable conditions. This occurred because there was a considerable redundancy and spatial complementary among the set of disturbances, such that any likely error/bias in one disturbance threshold could have been accounted for by any of the other disturbances.

**Variations in thresholds**: In the main body of the text, we used hard thresholds to calculate the date of unsuitable conditions. However, variations in these thresholds exist and some thresholds may be better defined than others, which may then affect our results. Furthermore, thresholds are not standardized on the same dependent response. To assess the magnitude of this caveat, we ran a sensitivity test in which dates of unsuitable conditions were re-calculated after a given threshold was set at values from 10% above and 10% below the used threshold. The results of this test are shown in S7 Fig. and indicate that lowering the tolerance thresholds of a single variable could cause the date of environmental unsuitability to be sooner than currently reported, suggesting that our results are conservative. This likely occurs because of the overlap of stressors that affects coral reefs (Fig. 3). That is, while a low threshold in one variable may prevent unsuitable conditions in that variable, overall unsuitable conditions may be driven by another of the disturbances studied. A combination of stressor thresholds that have been over- or under-estimated in the same direction could impact the resulting date of unsuitability, but the large number of tests necessary to properly represent every combination hindered the ability to include this.

**Recovery and adaptation**: Recovery to the impacts of a disturbance can come from the stressor diminishing thereby allowing the ecosystem to recover to its previous state, or by the stressor continuing to exceed thresholds and the species adapting to those new conditions. The possibility that reefs will recover even with the persistence of stressors is possible via species adaptation, but as it has been previously acknowledged, this option will be limited given the speed of environmental change [4,5]. This study also reveals the fast pace of changes, and short times for adaptation, in addition to the numerous stressors that coral reefs would have to adapt to. So, while our paper does not account for the potential adaptation of ecosystems, our results highlight the challenges of this evolutionary option for coral reefs. The other possibility for ecosystem recovery to disturbances is for the disturbances to diminish below their tolerance threshold in the future. To account for this possibility influencing our results, the date of unsuitable conditions was defined exclusively as the year after which the disturbance permanently exceeds the given threshold.

# **References**

1. Dixon AM, Forster PM, Heron SF, Stoner AMK, Beger M. Future loss of local-scale thermal refugia in coral reef ecosystems. PLOS Clim. 2022;1: e0000004. doi:10.1371/journal.pclm.0000004

2. Mora C. A clear human footprint in the coral reefs of the Caribbean. Proc R Soc B Biol Sci. 2008;275: 767–773. doi:10.1098/rspb.2007.1472

3. Darling ES, McClanahan TR, Côté IM. Combined effects of two stressors on Kenyan coral reefs are additive or antagonistic, not synergistic. Conserv Lett. 2010;3: 122–130. doi:10.1111/j.1755-263X.2009.00089.x

4. Donner SD, Skirving WJ, Little CM, Oppenheimer M, Hoegh-Guldberg O. Global assessment of coral bleaching and required rates of adaptation under climate change. Glob Change Biol. 2005;11: 2251–2265. doi:10.1111/j.1365-2486.2005.01073.x

5. Logan CA, Dunne JP, Ryan JS, Baskett ML, Donner SD. Quantifying global potential for coral evolutionary response to climate change. Nat Clim Change. 2021; 1–6. doi:10.1038/s41558-021-01037-2
