# Supplementary material for: Co-occurring anthropogenic stressors reduce the timeframe of environmental viability for the world’s coral reefs
Source: PLoS Biol. 2022 Oct 11;20(10):e3001821. doi: 10.1371/journal.pbio.3001821 (PMC9553053; doi:10.1371/journal.pbio.3001821)
Supplement: S1 Table — (DOCX) [file pbio.3001821.s003.docx]

## **S1 Table. Data sources.**

| **Variable** | **Dataset** |  | **Source** | **URL** |
| --- | --- | --- | --- | --- |
| DHW  (underlying data is SST) | empirical |  | OISST – NOAA | <https://www-esrl-noaa-gov.eres.library.manoa.hawaii.edu/psd/data/gridded/data.noaa.oisst.v2.html> |
|  | modeled |  | CMIP5 (variable name = tos) | <https://esgf-node.llnl.gov/search/cmip5/> |
|  |  |  |  |  |
| Ωarag |  | CO2SYS | NOAA/AOML | <https://cdiac.ess-dive.lbl.gov/ftp/co2sys/> |
|  | empirical | total alkalinity | GLODAP 2016 | <https://www.nodc.noaa.gov/ocads/oceans/glodap/GlopDV.html> |
|  |  | total dissolved inorganic carbon | GLODAP 2016 | <https://www.nodc.noaa.gov/ocads/oceans/glodap/GlopDV.html> |
|  |  | temperature | WOA09 – NOAA | <https://www.nodc.noaa.gov/OC5/WOA09/pr_woa09.html> |
|  |  | salinity | WOA09 – NOAA | <https://www.nodc.noaa.gov/OC5/WOA09/pr_woa09.html> |
|  |  | phosphate | WOA09 – NOAA | <https://www.nodc.noaa.gov/OC5/WOA09/pr_woa09.html> |
|  |  | silicate | WOA09 – NOAA | <https://www.nodc.noaa.gov/OC5/WOA09/pr_woa09.html> |
|  | modeled | salinity | CMIP5 (so) | <https://esgf-node.llnl.gov/search/cmip5/> |
|  |  | SST | CMIP5 ( tos) | <https://esgf-node.llnl.gov/search/cmip5/> |
|  |  | total phosphate | WOA18 – NOAA | <https://www.nodc.noaa.gov/OC5/woa18/woa18data.html> |
|  |  | total silicate | WOA18 – NOAA | <https://www.nodc.noaa.gov/OC5/woa18/woa18data.html> |
|  |  | total alkalinity | CMIP5 (talk) | <https://esgf-node.llnl.gov/search/cmip5/> |
|  |  | total dissolved inorganic CO2 | CMIP5 (dissic) | <https://esgf-node.llnl.gov/search/cmip5/> |
|  |  |  |  |  |
| population | empirical |  | GPW v1 | <https://sedac.ciesin.columbia.edu/data/set/popdynamics-global-pop-density-time-series-estimates/data-download> |
|  | model |  | HYDE 3.1 | <https://dataportaal.pbl.nl/downloads/HYDE/HYDE3.1/> |
|  |  |  | IAM | <https://www.cgd.ucar.edu/iam/modeling/spatial-population-scenarios.html> |
|  |  |  |  |  |
| land use | model |  |  | <https://daac.ornl.gov/VEGETATION/guides/Land_Use_Harmonization_V1.html> |
|  | empirical |  | HYDE 3.2 | <https://themasites.pbl.nl/tridion/en/themasites/hyde/download/index-2.html> |
|  |  |  |  |  |
| storms | historic |  | IBTrACS | <https://www.ncdc.noaa.gov/ibtracs/index.php?name=ibtracs-data> |
|  | model |  | Emanuel, 2013 | <https://www.pnas.org/content/110/30/12219> |
|  |  |  |  |  |
| coral distribution |  | current coral | UNEP-WCMC | <https://data.unep-wcmc.org/datasets/1> |
|  |  | rocky reefs |  | <https://knb.ecoinformatics.org/view/doi:10.5063/F19Z92TW> |

* No gridded population datasets were available covering the full range of 1850-2100. Instead, we used two datasets independently covering the historical 1850-2005 range and the scenarios 2005-2100. This creates a disparity in the underlying raw data that results in a variation in the amount of unsuitable coral reef sites.
