# Supplementary material for: Co-occurring anthropogenic stressors reduce the timeframe of environmental viability for the world’s coral reefs
Source: PLoS Biol. 2022 Oct 11;20(10):e3001821. doi: 10.1371/journal.pbio.3001821 (PMC9553053; doi:10.1371/journal.pbio.3001821)
Supplement: S2 Table — (DOCX) [file pbio.3001821.s004.docx]

## **S2 Table. CMIP5 models.**

|  |  | SST (tos) | | | |  | Omega aragonite (dissic, so, talk) | | | |
| --- | --- | --- | --- | --- | --- | --- | --- | --- | --- | --- |
| model name |  | historic | RCP 2.6 | RCP 4.5 | RCP 8.5 |  | historic | RCP 2.6 | RCP 4.5 | RCP 8.5 |
| CanESM2 |  | 1 | 1 | 1 | 1 |  | 1 | 1 | 1 | 1 |
| CMCC-CESM |  | 1 |  |  | 1 |  |  |  |  |  |
| CMCC-CM |  | 1 |  | 1 | 1 |  |  |  |  |  |
| CMCC-CMS |  | 1 |  | 1 | 1 |  |  |  |  |  |
| GISS-E2-H |  | 1 | 1 | 1 | 1 |  |  |  |  |  |
| GISS-E2-H-CC |  | 1 |  | 1 | 1 |  |  |  |  |  |
| GISS-E2-R |  | 1 | 1 | 1 | 1 |  |  |  |  |  |
| GISS-E2-R-CC |  | 1 |  | 1 | 1 |  |  |  |  |  |
| HadGEM2-AO |  | 1 | 1 | 1 | 1 |  |  |  |  |  |
| HadGEM2-CC |  | 1 |  | 1 | 1 |  | 1 |  | 1 | 1 |
| HadGEM2-ES |  | 1 | 1 | 1 |  |  | 1 | 1 | 1 |  |
| inmcm4 |  | 1 |  | 1 | 1 |  |  |  |  |  |
| MIROC-ESM |  | 1 | 1 | 1 | 1 |  | 1 | 1 | 1 | 1 |
| MIROC-ESM-CHEM |  | 1 | 1 | 1 | 1 |  | 1 | 1 | 1 | 1 |
| MPI-ESM-LR |  | 1 |  |  |  |  | 1 |  |  | 1 |
| MRI-CGCM3 |  | 1 | 1 | 1 | 1 |  |  |  |  |  |
|  |  |  |  |  |  |  |  |  |  |  |
| total |  | 16 | 8 | 14 | 14 |  | 6 | 4 | 5 | 5 |
