# Supplementary material for: Co-occurring anthropogenic stressors reduce the timeframe of environmental viability for the world’s coral reefs
Source: PLoS Biol. 2022 Oct 11;20(10):e3001821. doi: 10.1371/journal.pbio.3001821 (PMC9553053; doi:10.1371/journal.pbio.3001821)
Supplement: S1 Fig — (DOCX) [file pbio.3001821.s005.docx]

## **S1 Fig. Coral & rocky reef locations.**

All coral and rocky reef locations evaluated in this study. The data underlying this Figure can be found in <https://zenodo.org/record/7055724>. Basemap provided by Esri [1].


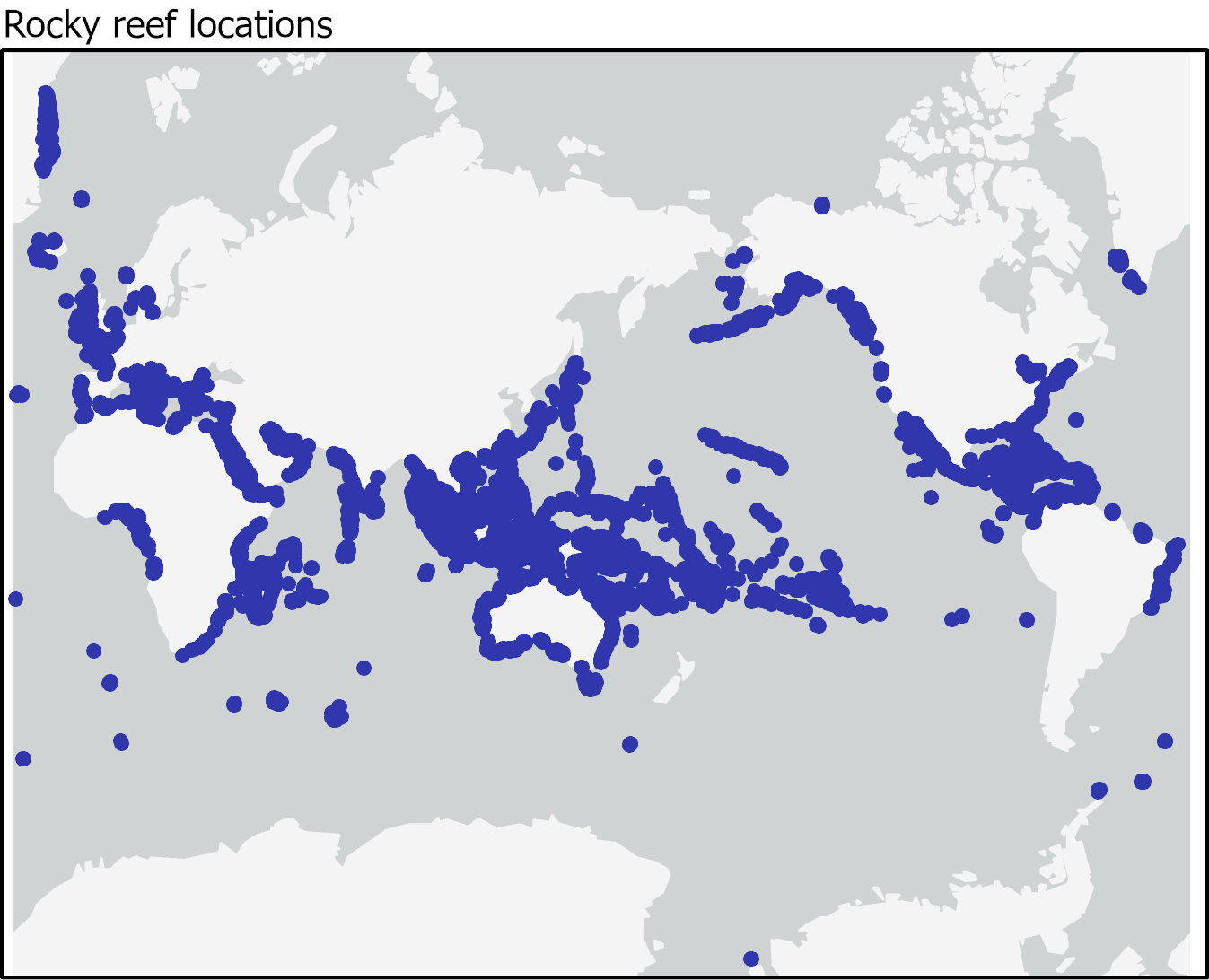


# **References**

1. Esri. “Light Gray Canvas Base” [basemap]. Available: https://basemaps.arcgis.com/arcgis/rest/services/World_Basemap_v2/VectorTileServer
