# Supplementary material for: Co-occurring anthropogenic stressors reduce the timeframe of environmental viability for the world’s coral reefs
Source: PLoS Biol. 2022 Oct 11;20(10):e3001821. doi: 10.1371/journal.pbio.3001821 (PMC9553053; doi:10.1371/journal.pbio.3001821)
Supplement: S5 Fig — (DOCX) [file pbio.3001821.s009.docx]

In S2-S6 Figs, global maps of the year of unsuitable conditions for coral reef ecosystems due to a given unsuitable environmental variable under three case scenarios (RCP2.6-SSP1, RCP4.5-SSP2, RCP8.5-SSP5) are depicted. Maps are visualized on a 0.5 degree grid. The data underlying this Figure can be found in <https://zenodo.org/record/7055724>. All basemaps provided by Esri [1].

## **S5 Fig. Date of unsuitable conditions by land use.**


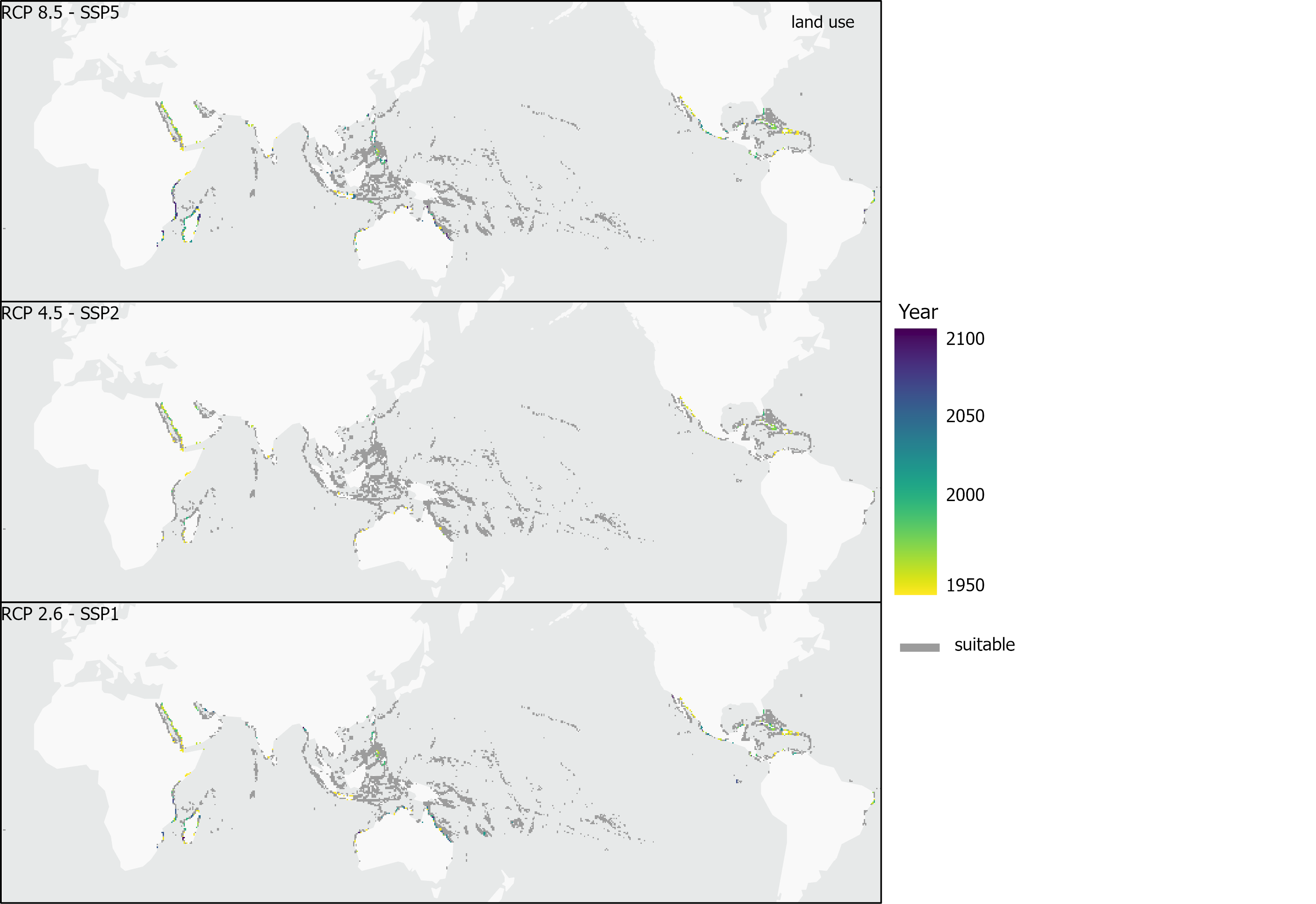


# **References**

1. Esri. “Light Gray Canvas Base” [basemap]. Available: https://basemaps.arcgis.com/arcgis/rest/services/World_Basemap_v2/VectorTileServer
