# Supplementary material for: Co-occurring anthropogenic stressors reduce the timeframe of environmental viability for the world’s coral reefs
Source: PLoS Biol. 2022 Oct 11;20(10):e3001821. doi: 10.1371/journal.pbio.3001821 (PMC9553053; doi:10.1371/journal.pbio.3001821)

## **S7 Fig. Sensitivity analysis to variations in threshold values.**

The effect of varying thresholds on the overall date of unsuitable conditions was assessed through a sensitivity analysis. The sensitivity test was conducted by calculating new threshold values that were 5% and 10% above and below the used threshold (see used thresholds in Table 1), then calculating the overall date of unsuitable conditions under RCP4.5 – SSP2 with just one variable threshold modified at a time. The median global dates of unsuitable conditions under each of these threshold modifications are displayed below. The data underlying this Figure can be found in <https://zenodo.org/record/7055724>.


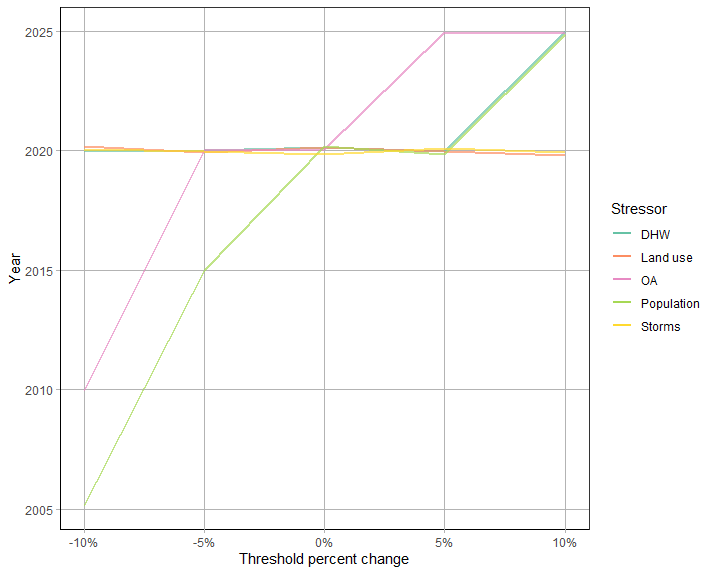

Supplement: S7 Fig — (DOCX) [file pbio.3001821.s011.docx]
